# *In silico* studies of ASEM analogues targeting α7-nAChR and experimental verification†

**DOI:** 10.1039/d0ra10435c

**Published:** 2021-01-21

**Authors:** Yang Zhou, Guanglin Kuang, Junhao Li, Christer Halldin, Agneta Nordberg, Bengt Långström, Yaoquan Tu, Hans Ågren

**Affiliations:** School of Pharmacy, Jinan University Guangzhou 510632 China; Department of Physics and Astronomy, Uppsala University Box 516 SE-751 20 Uppsala Sweden; Division of Theoretical Chemistry and Biology, School of Biotechnology, Royal Institute of Technology (KTH), AlbaNova University Center S-106 91 Stockholm Sweden; Karolinska Institutet, Department of Clinical Neuroscience, Centre for Psychiatric Research 171 76 Stockholm Sweden; Nordberg Translational Molecular Imaging Lab, Division of Clinical Geriatrics, Center for Alzheimer Research, Department of Neurobiology, Care Sciences and Society, Karolinska Institutet 141 84 Stockholm Sweden; Theme Aging Karolinska University Hospital S-141 86 Stockholm Sweden; Department of Chemistry, Uppsala University SE- 751 23 Uppsala Sweden; College of Chemistry and Chemical Engineering, Henan University Kaifeng Henan 475004 P.R. China

## Abstract

The α7 nicotinic acetylcholine receptor (α7-nAChR) is implicated in a variety of neurodegenerative and neuropsychiatric disorders, such as Alzheimer's disease (AD) and schizophrenia. The progress of these disorders can be studied using positron emission tomography (PET) with radiotracers for α7-nAChR. [^18^F]ASEM and [^18^F] *para*-ASEM (also referred to as [^18^F]DBT-10) are novel and potent α7-nAChR PET radiotracers which have successfully been used in human subjects and nonhuman primates, though further improvement of them is still a pressing task in the community of neurodegeneration research. In this work, we demonstrate the use of modern *in silico* techniques to predict the binding modes, binding strengths, and residence times for molecular PET tracers binding to proteins, using ASEM and DBT-10 as a showcase of the predictive and interpretational power of such techniques, in particular free energy perturbation theory. The corresponding compounds were synthesized and further tested by *in vitro* binding experiment for validation. Encouragingly, our *in silico* modeling can correctly predict the binding affinities of the ASEM analogues. The structure–activity relationships for the *ortho*- and *para*-substitutions are well explained at the atomistic level and provide structure-based guiding for the future development of PET tracers for α7-nAChR. A discussion is presented on the complementary use of *in silico* rational methods based on atomic and electronic principles for *in vitro* characterization of PET tracers.

## Abstract 2

The *in silico* binding free energies of the ASEM analogues targeting α7-nAChR are in line with the inhibition obtained from *in vitro* binding assays.

## Graphical abstract

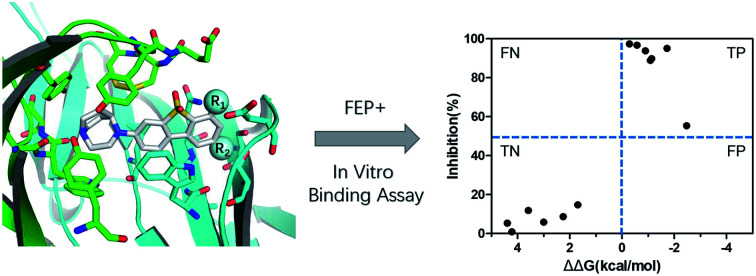

## Introduction

Nicotinic acetylcholine receptors (nAChRs) are neurotransmitter-gated ion channels that are widely distributed in the central nervous system (CNS), peripheral nervous system, and various non-neuronal cells. In human CNS, nAChRs are pentamers composed of various combinations of twelve different subunits (α2–α10 and β2–β4), with the homo-pentameric α7-nAChR (all α7 subunit) and hetero-pentameric α4β2-nAChR (α4 and β2 subunits in a 2 : 3 or 3 : 2 ratio) being predominant.^1^ α7-nAChR is mainly expressed in the hippocampus and cortex regions which are involved in cognition and memory.^2^ Clinical studies have demonstrated that α7-nAChR is implicated in a variety of neurodegenerative and neuropsychiatric disorders such as Alzheimer's disease (AD) and schizophrenia. A reduced density of α7-nAChR has been seen post-mortem in patients with Alzheimer's disease (AD) and schizophrenia.^3^ Therefore, α7-nAChR is assumed to be an important target to treat neurodegenerative and neuropsychiatric diseases. Many agonists and positive allosteric modulators (PAM) of α7-nAChR are currently under development worldwide in both industry and academia.^4^

**Fig. 1:**
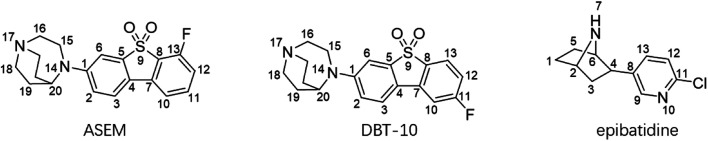
Molecular structure of ASEM, DBT-10, and epibatidine.

The mechanism and progress of CNS diseases related to α7-nAChR can be studied with positron emission tomography (PET), which is an advanced technique to visualize and quantify receptors and their occupancy by neurotransmitters and drugs in human brains. *In vivo* PET studies are superior to post-mortem studies in that it is non-invasive and can be used to study the early stages of CNS diseases. PET studies can also facilitate the development of drugs targeting α7-nAChR by measuring receptor occupancies and dose–response relationships. However, the development of PET radioligands for α7-nAChR is challenging because α7-nAChR exhibits very low density in the CNS (0.3–15 fmol mg^−1^ protein in humans).^5^ A successful PET radioligand for α7-nAChR must exhibit a combination of picomolar binding affinity, high specificity (BP_ND_ > 1), and other essential characteristics. [^11^C] CHIBA-1001 is the first α7-nAChR PET radiotracer to be studied in human subjects but exhibits low affinity (*K*_d_ ∼ 46 nM) and low non-displaceable binding potential (BP_ND_ < 1).^6,7^ In the past decade, many new PET radioligands have been developed for the imaging of α7-nAChR, such as [^11^C]A-582941, [^11^C] (*R*)-MeQAA, and [^18^F]NS14492.^8–10^ However, all these PET radiotracers failed in *in vivo* imaging applications in the human brain due to factors like low affinity or low specificity. [^18^F]ASEM developed by Gao and Horti *et al.* is a novel PET tracer that has a higher affinity (*K*_d_ ∼ 0.4 nM) and non-displaceable binding potential (BP_ND_ ∼ 5) for α7-nAChR.^11,31^ It has successfully been used for human subjects and opens new horizons for studying α7-nAChR in living human brains.^12^ [^18^F]DBT-10 (referred to as [^18^F]*para*-ASEM) was developed as a part of the development of [^18^F]ASEM (Fig. 1). DBT-10 and ASEM are based on the same dibenzothiophene scaffold, differing in the position of the fluoro-substituent. The *in vitro* binding affinity values of ASEM and DBT-10 are contradictory under different experimental conditions. Assays using rat cortical membranes with [^125^I] α-bungarotoxin showed that a higher binding affinity of ASEM than DBT-10, while assays using cloned human α7-nAChR stably expressed in SH-SY5Y cells with [^3^H]methyllycaconitine showed the binding affinity of DBT-10 is slightly higher than ASEM. While such contradictory in binding affinity is not unusual in competition binding assays with different radioligands, it can affect the selection of the candidate for further *in vivo* evaluation. In the early research, only ASEM was selected for further *in vivo* evaluations. Later studies show DBT-10 has highly similar kinetic properties as ASEM in nonhuman primates and it has also received considerable attention for PET imaging.^13^

**Fig. 2:**
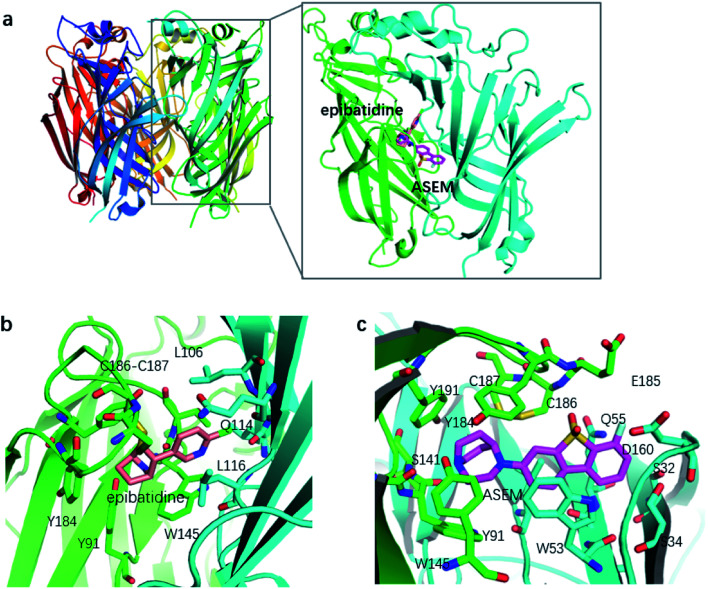
(a) Structures of α7-AChBP and its binding site, (b) the binding mode of epibatidine with α7-AChBP, and (c) the binding mode of ASEM with α7-AChBP. Epibatidine and ASEM are shown in thick stick mode while other residues in thin stick mode. Non-polar hydrogens are not shown for clarity.

Despite the clinical importance of α7-nAChR, limited knowledge is available regarding its structure. Until recently the structural information of α7-nAChR was mainly inferred from the acetylcholine binding protein (AChBP) from snails *Lymnaea stagnalis* (Lys-) and *Aplysia californica* (Apc-).^14^ These structures provide valuable information about the general configuration and organization of α7-nAChR. However, they have limited usage in the investigation of the function of α7-nAChR, especially the ligand binding profile, due to the low sequence identities with α7-nAChR. In 2011, a chimera structure of Lys-AChBP and the extracellular domain of the human α7-nAChR was determined (Fig. 2).^15^ This chimera structure (termed as α7-AChBP in this work) has a sequence identity as high as 64% with native α7-nAChR and is by far one of the best structural models for α7-nAChR. More importantly, the ligand-binding site and surrounding areas of this receptor chimera are lined entirely by α7-nAChR residues, making the chimera structure a good model to study the binding profile of α7-nAChR with its ligands.^15^

In this work, the binding modes of ASEM and DBT-10 are studied and their binding affinity differences were revealed by free energy perturbations calculations *via* the FEP+ module of Schrödinger Inc.^16^ A series of analogues of ASEM with different *ortho*- and *para*-substitutions were designed. They were selected based on the labeling potential using various ^11^C-methylation and/or ^11^C-carbonylation reactions.^17,18^ The binding affinities of the analogues were estimated by FEP+ based on the binding modes of ASEM and DBT-10. Then, the analogues were synthesized and further tested by *in vitro* experiment. The predicted binding free energy values are in good agreement with experimental results. Analysis of the binding modes in conjunction with the experimental data can help us understand the structure–activity relationship of *ortho*- and *para*-substitutions at atomistic detail. The modeling work provides us the structure-based modification directions for improving the properties of ASEM and diverse the PET candidates for the imaging of α7-nAChR.

## Results

### Binding mode of ASEM with α7-AChBP

Like the crystallized ligand epibatidine (see Fig. 2), ASEM has a diazobicyclic head group and is protonated under physiological conditions. However, the diazobicyclic head group of ASEM is bulkier than the counterpart of epibatidine, which should have an impact on its binding with the receptor. Besides, the dibenzothiophene ring of ASEM is also much bigger than the pyridine ring of epibatidine. These two structural differences make epibatidine and ASEM have different potency profiles, namely, epibatidine is an agonist whereas ASEM is an antagonist. This is consistent with the general knowledge that α7-nAChR antagonists such as methyllycaconitine(MLA) and α-bungarotoxin tend to be much bulkier than the agonists such as nicotine and acetylcholine. In a standard docking procedure where the receptor was held rigid, ASEM could not be docked properly, with a less favorable docking score (−4.53 kcal mol^−1^), which is most probably due to the small size of the binding pocket occupied by epibatidine (docking score: −8.84 kcal mol^−1^). However, with the induced fit docking (IFD) procedure, ASEM could be docked to the binding site with a much more favorable docking score (−10.8 kcal mol^−1^). This is reasonable because ASEM is bulkier and would need more space for binding. The residues relaxed most significantly are Trp53, Tyr91, Trp145, Tyr184, Cys186, Cys187, and Tyr191. The tip nitrogen (N1, p*K*_a_ ∼ 9.6) of ASEM is protonated under physiological conditions and has cation–π interactions with the aromatic rings of Tyr91, Trp145, Tyr184, and Try191. These cation–π interactions are believed to be important for the affinity of α7-nAChR ligands. The protonated nitrogen also forms a hydrogen bond with the backbone oxygen of Trp145 (Fig. 2c). Besides, the diazobicyclic group has extensive van der Waals interactions with the side chains of Tyr91, Trp145, Tyr184, and Try191. Glide docking score decomposition of residues around the binding site shows that van der Waals interactions from Tyr91, Trp145, and Tyr191 has a major contribution to the docking score, which helps to stabilize ASEM in the binding site. The most significant difference between the binding modes of epibatidine and ASEM was seen in the aromatic tail part (Fig. 2b and c). For epibatidine, the chloro-pyridine ring lies in the cavity formed by Leu106, Gln114, and Leu116 and has van der Waals or hydrophobic interactions with these residues. Besides, the chlorine atom is thought to have halogen-bond interaction with the backbone oxygen atom of Gln114, which also supports the binding of epibatidine. However, for ASEM, the dibenzothiophene ring is too big to fit into the site originally occupied by the pyridine ring of epibatidine. As a result, it adopts a different orientation and lies in the cavity on the other side which is formed by Ser34, Leu36, Trp53, Asp160, Gly163, Tyr184, Glu185, Cys186, and Cys187 (Fig. 2c). The dibenzothiophene ring is clenched by van der Waals interactions with Glu185, Cys186, and Cys187 from loop C (residues 180–193) on one side and π–π stacking interaction with Trp53 from the complementary subunit on the other side (Fig. 2c). Ser34, Leu116, and Asp160 also have some contact with the dibenzothiophene ring. The fluorine and oxygen atoms of ASEM point towards the solvent and do not have much interaction with surrounding residues. With induced-fit docking, we managed to produce a reasonable docking mode of ASEM with α7-AChBP, which will be used as the starting point for subsequent analysis.

**Fig. 3:**
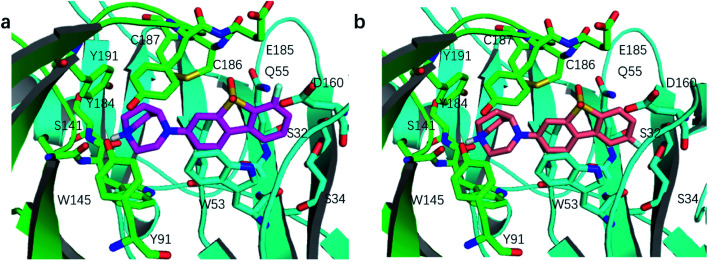
The predicted binding mode of ASEM (a) and DBT-10 (b).

### Comparison of the binding modes of ASEM and DBT-10

We compared the difference in the binding mode between ASEM and DBT-10 (Fig. 3). The binding mode of ASEM suggests that the fluorine atom of ASEM points towards the solvent and does not have much interaction with the surrounding residues, while the fluorine atom of DBT-10 is predicted to point toward the inside of the pocket. The fluorine atom of DBT-10 occupies the hydrophilic region near Ser32 and Ser34. The formation of extra interactions between the fluorine atom of DBT-10 and protein residues increases the binding affinity. Free energy perturbation calculations by FEP+ shows that DBT-10 has a lower relative binding free energy than ASEM (ΔΔ*G* = −0.24 ± 0.02 kcal mol^−1^), indicating that DBT-10 has a slightly higher binding affinity than ASEM. The higher binding affinity of DBT-10 is in agreement with the *in vitro* experiment using the human α7-nAChR and is in contradiction to the result from rat cortical membranes.^13^ This may be due to that the chimera structure of AChBP we use in the FEP calculations has a higher identity to the human α7-nAChR.

### *Ortho*- and *para*-substitutions of ASEM and binding affinities prediction

**Fig. 4:**
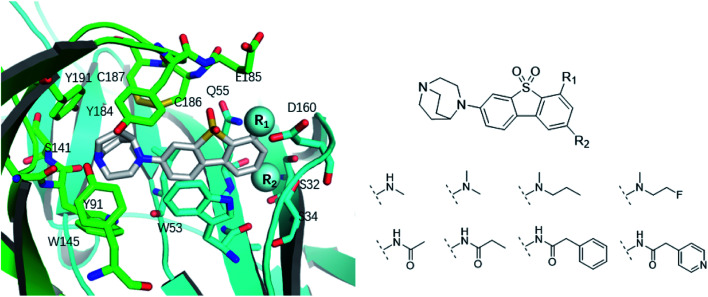
Binding mode of ASEM with α7-AChBP and the structure of ASEM analogues.

The binding modes of ASEM and DBT-10 indicate that the *ortho*-substitution points towards the solvent (R_1_ in Fig. 4), while the *para*-substitution points towards the hydrophilic region between Ser32 and Ser34 (R_2_ in Fig. 4, right). To explore the effect of *ortho*- and *para*-substitutions on binding, we designed a series of ASEM analogues (Fig. 4, right). Chemical groups with different sizes and properties, such as *N*-methyl, *N*,*N*-dimethyl, *N*-methyl-*N*-propyl, acetyl, propionyl, and phenylpropionyl, were designed to substitute the *ortho*-fluorine on ASEM and *para*-fluorine on DBT-10.

Free energy perturbation calculations were carried out for the designed ASEM analogues. The relative binding free energies of the analogues relative to ASEM are shown in Table 1. Compared with ASEM, the substitutions on R_1_ position all have a negative ΔΔ*G*, which means that the modifications at this position can remain or improve the activity of the compounds. The substitutions on R_2_ position result in a positive ΔΔ*G* relative to ASEM, which means that these compounds could have lower binding affinities than ASEM. For the R_2_ substitution, even a small substituent such as *N*-methyl can cause a positive ΔΔ*G*. The ΔΔ*G* is higher when substitutions are large groups such as propionyl and phenylpropionyl.

**Table 1:** Relative free energies and physiochemical properties of ASEM analogues

Cpd ID	R_1_	R_2_	ΔΔ*G*^a^ (kcal mol^−1^)	Inhibition (%)	Alog *P*^b^	Plasma protein binding	BBB c	P-gp substrate	Residence time^d^ (ns)
ASEM	F	H	0	94.2	2.9	1.19	+	+(0.65)	53.4
1	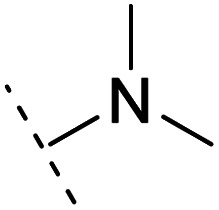	H	−0.3	97.8	2.9	1.06	+	+(0.74)	75.6
2	H	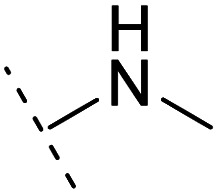	+1.7	14.8	2.8	1.00	+	+(0.68)	30.0
3	H	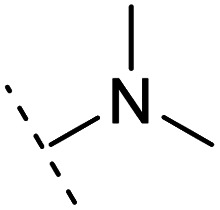	+1.5	11.9	2.9	1.04	+	+(0.66)	—
4	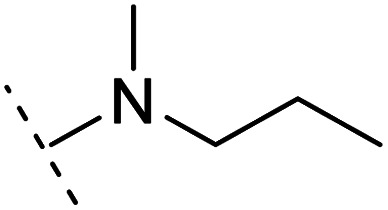	H	−1.7	95.1	3.6	1.06	+	+(0.79)	87.3
5	H	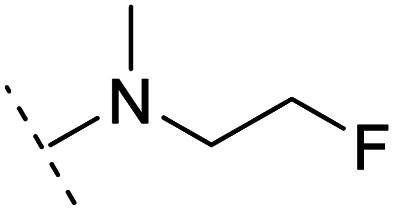	1.6	5.9	3.2	1.05	+	+(0.73)	—
6	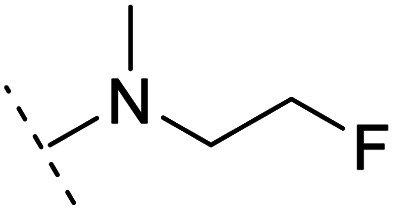	H	−0.6	96.7	3.2	1.03	+	+(0.78)	51.2
7	H	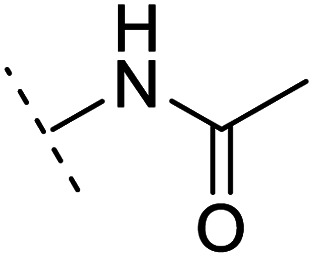	+2.3	8.7	2.7	1.05	+	+(0.78)	—
8	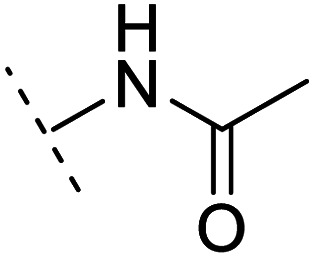	H	−1.1	88.9	2.7	1.07	+	+(0.81)	58.3
9	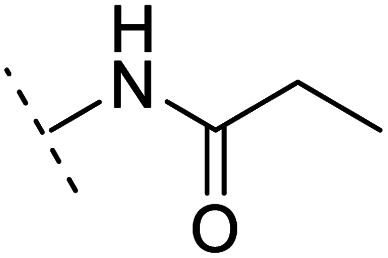	H	−0.9	93.8	2.5	1.08	+	+(0.81)	—
10	H	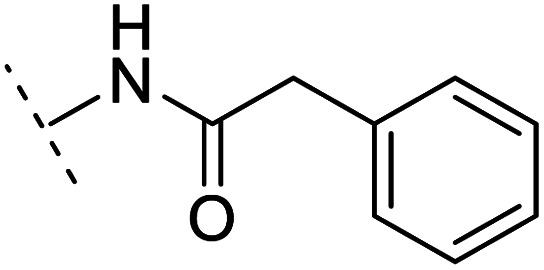	+4.2	0.9	4.0	1.21	+	+(0.71)	14.4
11	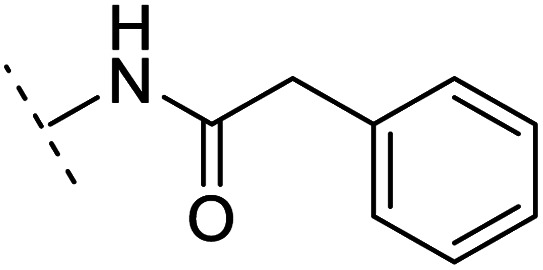	H	−1.1	89.8	4.0	1.28	+	+(0.69)	14.2
12	H	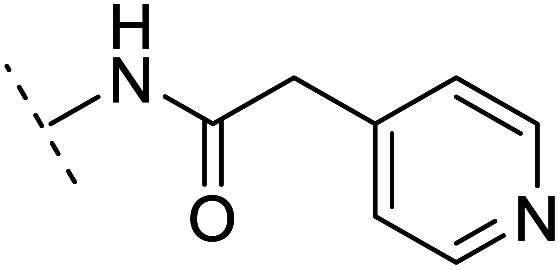	+4.4	5.4	3.4	1.10	+	+(0.72)	—
13	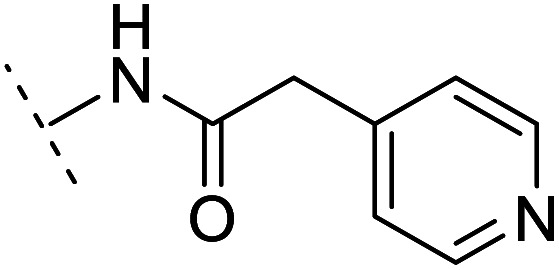	H	−2.5	55.4	3.4	1.13	+	+(0.74)	40.8

a The relative free energy is calculated with ASEM as the reference. The standard errors are in the range of 0.1 to 0.5 kcal mol^−1^.

b Alog *P* is calculated using Schrodinger.

c BBB and P-gp were predicted by our in-house machine learning tools based on cheminformatics using Python, sklearn and rdkit.

d The residence time was calculated with potential scaled MD simulations. The standard errors are in the range of 1 to 5 ns.

In addition to changing the binding ability of the compound, using substituents to adjust the physical and chemical properties of the compound is also an important part on the development of PET radioligands for α7-nAChR. The physiochemical properties were calculated and are shown in Table 1. All designed compounds can pass the blood–brain barrier and are P-gp substrate in the prediction. The log *P* increases when the substituents are lipophilic groups. The same substitution at R_1_ or R_2_ has little effect on the log *P*, plasma protein binding, blood–brain barrier permeability, or whether it is a P-gp substrate.

### *In vitro* validation and explanations for the activity from a structure of view

**Fig. 5:**
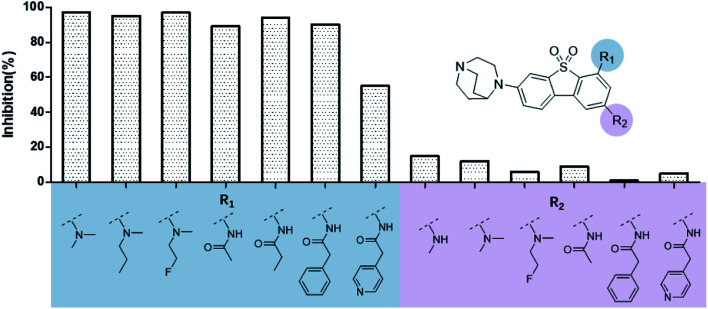
Histogram for inhibition of ASEM analogues with substitutions at the R_1_ and R_2_ positions.

**Fig. 6:**
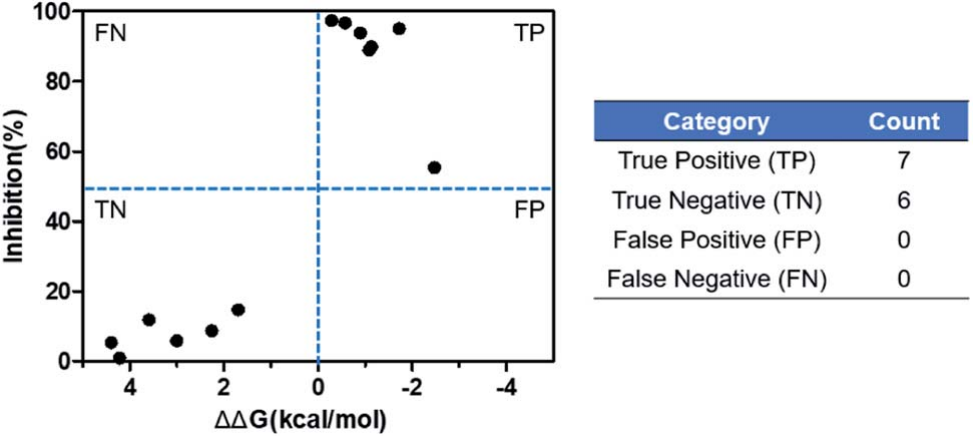
Comparison of the *in silico* binding free energy difference calculated by FEP+ and *in vitro* inhibition.

The ASEM analogues with substitutions at the R_1_- and R_2_-positions were synthesized and tested by *in vitro* experiment using recombinant human α7-nAChR expressed in SH-SY5Y cells with [^125^I]-α-bungarotoxin (see Methods). The inhibition at a test concentration of 10 μM are shown in Fig. 5. Substitutions at R_1_ position can retain the binding affinity with inhibition above 90%. At R_1_, even the bulky substituents such as propionyl and phenylpropionyl can remain the inhibition as high as 90%. At the R_1_ position, only the pyridine ring substituent has inhibition of 55%. Substitutions at R_2_ position almost abolished the activity of the analogues. The inhibition of substituents at R_2_ position is less than 20%. The small groups such as *N*-methyl and *N*,*N*-dimethyl are slightly better with the inhibition of 15% and 12%, respectively. The experimental results are in good agreement with the previous ΔΔ*G* predictions which demonstrates that the calculated binding affinities for the R_1_-analogues are more favorable than those for the R_2_-analogues. Note that a completely linear relationship is not expected since there is an underlying dose concentration dependence. As shown in Fig. 6, with a cutoff of 50% inhibition, the true positive rate (TPR or sensitivity) and false-positive rate (FPR or fall-out) are 100% and 0%, respectively.

**Fig. 7:**
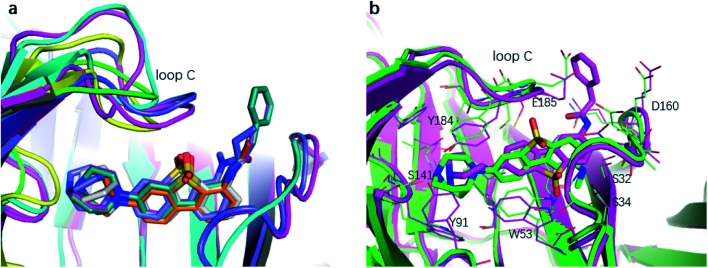
The binding mode of R_1_- and R_2_-analogues. (a) Representative structures of R_1_-analogues. (b) Representative structures of R_2_-analogues.

The binding affinity differences of the R_1_-and R_2_-analogues can be explained by their binding modes in the protein. As shown in Fig. 7a, the R_1_-position is close to loop C of the binding pocket. Loop C is flexible and showed the open-closed mechanism in previous studies.^19^ For substitutions at R_1_ position, loop C can open up and offer extra spaces for the chemical groups. Therefore, R_1_-analogues can maintain the binding affinity even when the substituents are as large as propionyl and phenylpropionyl. In contrast, the R_2_-position is deeply buried and has close interactions with nearby residues Ser32 and Ser34. Substituents at R_2_ position can lead to steric conflict between the protein and ligand. As shown in Fig. 7b, the *N*-methyl substituent at the R_2_ position leads a steric conflict to Ser32 and Ser34. When the substituent is large, such as phenylpropionyl group, there is no more space for such a group, and the dibenzothiophene ring flips to let the phenylpropionyl group point towards the solvent *via* the egress portal between Glu185 and Asp160. The π–π stacking interaction between the dibenzothiophene ring and Trp53 is therefore lost. The steric conflict also changes the position of the diazobicyclic ring. The hydrogen bond between the protonated nitrogen of the diazobicyclic ring and the backbone oxygen of Trp145 is weakened. The cation–π interactions between the diazobicyclic ring and the aromatic rings of Tyr91, Trp145, Tyr184, and Try191 are also weakened because of the conformational changes. As a result, R_2_-substitutions are generally less favorable than the R_1_-substitutions. R_1_-position substitutions are thus quite tolerant of bulky chemical groups.

### Prediction of the unbinding kinetics

**Fig. 8:**
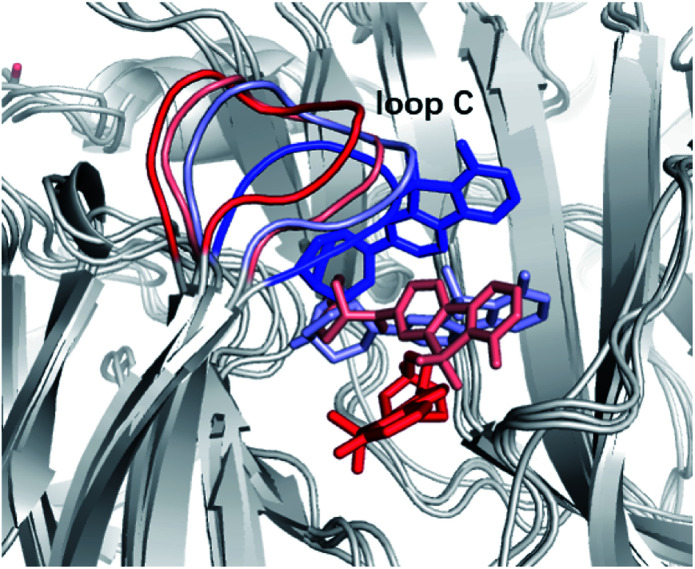
Time evolutions of ASEM and loop C from red (*t* = 0) to blue (unbound) during the unbinding process.

The measurements of kinetic parameters for ligands cannot disclose the way the tracer leaves the protein receptor nor give the information on the molecular determinants that dictate the dissociation process.^27^ That leaves out the key interactions and conformational changes essential for structural optimization of tracers concerning the kinetic properties. Luckily, enhanced sampling techniques have emerged recently as effective tools for studying unbinding kinetics of protein–ligand systems at the atomistic level. However, for practical computational studies, it is necessary to consider the fact that the ligand unbinding processes are strongly coupled to protein conformational changes and that there may be hidden degrees of freedom to disclose. This poses still a great challenge for sampling. In ref. 27 it was shown how potential scaled molecular dynamics (sMD) and infrequent metadynamics (InMetaD) simulation techniques could be combined to successfully reveal the unbinding mechanism of ASEM from the chimera structure of the α7-AChBP receptor. It was possible to utilize these simulation techniques to pinpoint the important role of certain structural units in the unbinding process, and in particular, to identify that the motion of “loop C” (see Fig. 8) could be critical for the ASEM unbinding process. Here one could follow the progression of opening and closing of this loop as the most relevant slow degree of freedom for the unbinding. One could furthermore see that there is more than one metastable state involved in the unbinding process and that the rate-limiting steps, and associated transition state structures, are associated with the interactions between the residues of the binding pocket and the sulfone group of the ASEM tracer. With the applied simulation techniques, it is possible to sample the slow conformational rearrangement of a fibrillar-tracer system occurring at the timescale beyond seconds, which thus makes it possible to consider these slow conformational changes which are critical to the ligand unbinding process. Thus, with modern molecular dynamics techniques, the detailed mechanisms of the unbinding process can be revealed which paves the way for studying the unbinding kinetics of protein–ligand systems in general and for the optimization of new tracers towards certain receptors with maximum properties.

In addition to the binding affinities and binding modes, the unbinding kinetics of the tracer-receptor systems is thus of great importance for the design of tracers with the desired specificity. To explore the effect of R_1_-and R_2_-substitutions on the kinetics property of the tracers, we recall here sMD simulations of the residence time (*τ*) of the ASEM analogues as described in method section.^27^ The results are recapitulated in Table 1. We would like to point out that the estimated residence times are not real residence times. However, they can be used to rank the experimental residence times. In sMD simulations, the reference residence time of ASEM is predicted to be 53.4 ns, while compound 1 with *N*,*N*-dimethyl substituent (*τ* = 75.6 ns) and compound 4 with *N*-methyl-*N*-propyl at the R_1_-position (*τ* = 87.3 ns) have longer residence times although they have comparable binding affinity than ASEM. Compound 6 and 8 with *N*-fluoroethyl-*N*-methyl (*τ* = 51.2 ns) and acetyl substituent (*τ* = 58.3 ns) at R_1_-position share comparable residence time to ASEM. Substitutions at the R_2_ position are found to decrease the binding affinity and residence time, such as *N*-methyl (compound 2, *τ* = 30.0 ns) and phenylpropionyl (compound 10, *τ* = 14.4 ns). Large groups at R_1_-position, such as phenylpropionyl (compound 11, *τ* = 14.2 ns) and pyridylpropionyl (compound 13, *τ* = 40.8 ns) have shorter residence time than ASEM. Considering that compound 11 has a comparable binding affinity to that of ASEM, and that a compound with a shorter residence time is desired in the structural modification of ASEM analogues, compound 11 may be a better choice as PET tracer for α7-nAChR.

## Discussion

Owing to the importance of developing potent PET radioligands which can be used to study the roles of the α7 nicotinic acetylcholine receptor (α7-nAChR), to facilitate drug discovery and to monitor the progress of diseases related to α7-nAChR, molecular modeling methods have been used in this work to investigate the binding profile of [^18^F]ASEM (a promising PET radioligand) and α7-AChBP (a structural homologue of the extracellular domain of α7-nAChR). We studied the binding features of [^18^F]ASEM at the orthosteric site of α7-AChR. Several structural details of this binding are found to be important. The diazabicyclo[3.2.2]nonane ring has cation–π and extensive van der Waals interactions with Tyr91, Trp145, Tyr184, and Try191, which fixes [^18^F]ASEM tightly in the binding site. The dibenzothiophene ring turns to the other side of the pyridine ring of epibatidine (the crystallized agonist) and has van der Waals interactions with residues from loop C on one side and π–π stacking interaction with Trp53 of the complementary subunit on the other side. A series of ASEM analogues were calculated by FEP+ *in silico* and tested *in vitro*.

A second purpose of the present work was to demonstrate the general power of modern *in silico* approaches based on rational principles to predict the binding mode and binding energies of PET tracers to various protein structures, using Free Energy Perturbation Theory (FEP+) as the basic theoretical approach. Indeed, the consistency between *in silico* and, *a posteriori*, *in vitro* results indicates that FEP+ can accurately predict the binding free energy difference of ASEM analogues. This work thus indicates that the FEP+ utility as implemented in the Schrödinger suite of programs can greatly facilitate the development of α7-nAChR PET tracers using rational drug design strategies.

In addition to the focus on binding modes and energies, we shortly also reviewed some results on the kinetics of the [^18^F]ASEM unbinding from a chimera structure α7-nAChR as this is just as an important factor for the evaluation of tracer performance. This information is required to understand how long time it stays there (the residence time). We refer to our recent works for details on this, also very important, aspect of tracer design.^27^ We furthermore note that the techniques for binding modes and kinetics also can be used not only to explore the competitive binding of tracers for a given protein target and the competitive binding sites for a given tracer – protein pair but also to distinguish binding for a given tracer and different protein targets, which is a crucial aspect to design a tracer with sufficient selectivity on top of its efficiency.

As a final note, we emphasize that the validation between *in silico* and results from measurements have been conducted using *in vitro* binding assays. Going to *in vivo* a whole new situation appears for the modeling, as now factors like blood–brain-barrier (BBB) penetration, membrane protein binding (PgP) and lipophilicity, enter into the evaluation of the potency of tracers. For these factors, we see a great future for the almost explosive development of artificial intelligence in medicine, in particular using machine learning or deep learning methods. Indeed, we have recently been able to show that the *in silico* rational approaches can greatly be complemented into a full simulation chain going from atom to man for *in silico* predictions of PET tracers, including ASEM tracers. Such a chain needs several validation points in order to proceed in a meaningful way. This will be the subject for an upcoming publication and then including the preparation and further validation of the ^18^F or ^11^C-labelled candidates.

## Methods

### Molecular docking

In a standard molecular docking study, the receptor is held rigid, and the ligand can change its position and conformation freely. However, this procedure is problematic when the ligand to be docked is rather different from the crystallized one in shape or size. In reality, the receptor structure will undergo side-chain or backbone movements upon ligand binding to conform to the shape of the ligand, a process known as induced fit. The induced-fit docking (IFD) workflow of Schrödinger implements this idea through a combination of Glide and Prime jobs, which account for the conformational changes of the ligand and receptor, respectively.^19,20^ In this work, the crystal structure of the α7-AChBP chimera (PDB code 3SQ6)^15^ was used as the protein target. Before docking, the crystal structure was prepared with the protein preparation workflow of Schrödinger, where the hydrogen atoms were added and optimized, and the bond order was fixed. For the glide docking procedure, the centroid of the crystalized ligand epibatidine was chosen as the grid centre and the residues within 20 Å of it are treated as binding pocket. A van der Waals scaling factor of 0.5 was used for both receptor and ligand. In the induce fit docking process, protein residues within 5 Å of the ligand were optimized by prime. The glide standard precision (SP) scoring function was adopted to rank the optimized docking poses. The a7-AChBP/ASEM complex with the most favourable binding energy was chosen for subsequent analysis. The radionuclide fluorine-18 of ASEM, which is used in PET studies, is not indicated hereafter unless otherwise specified, as radiation is supposed not to affect the binding with a7-AChBP.^26^

### Free energy calculation using FEP+

The FEP+ utility of Schrödinger was used to calculate the free energy differences of ASEM analogues.^16^ The OPLS3 force field was used to describe the protein and ligands.^21^ Ligand atomic partial charges are computed *via* the CM1A-BCC.^22^ The REST (replica exchange with solute tempering) algorithm^23^ has been incorporated using Desmond as the MD engine. 2 Tesla K80 GPUs are used for FEP calculations. LOMAP mapping algorithm^24^ was used to set up the calculations and perturbation pathways. Fig. S1† illustrates the thermodynamic cycle used for calculating the binding free energy difference (ΔΔ*G*) between an ASEM analogue and ASEM, where arrows A and B represent the perturbation pathways. The maximum common substructure (MCS) between any pair of compounds is generated and their similarity is measured. Then ligand pairs with high similarity scores are connected by edges, where each edge represents one FEP calculation that will be performed between the two ligands. The systems of α7-AChBP with ASEM analogues were first relaxed and equilibrated using the default Desmond relaxation protocol. The whole system with the solute molecules restrained to their initial positions was first minimized using the Brownie integrator and then simulated at 10 K using an NVT ensemble followed by an NPT ensemble. After that the system was simulated at room temperature using the NPT ensemble with the restraints retained. Then the whole system without any restraint was simulated at room temperature using the NPT ensemble for 240 ps followed by the production simulation. A total of 12 λ windows were used for all the FEP/REST calculations. The production stage lasted 5 ns for both the complex and the solvent simulations using NPT ensemble conditions. Replica exchanges between neighboring *λ* windows were attempted every 1.2 ps. The Bennett acceptance ratio method (BAR) was used to calculate the free energy.^25^ Errors were estimated for each free energy calculation using both bootstrapping and the BAR analytical error prediction.

### Potential scaled MD simulations

The sMD simulations are carried out in line with the previous study.^27^ In brief, we employed ff99SB-ildn and GAFF force filed for the protein and the ligands, respectively. The restrained electrostatic potential-derived charges were used for ASEM with the electrostatic potential calculated at the Hartree–Fock level with the 6-31G* basis set using Gaussian 09.^28^ The TIP3P^29^ water model was used to solvate the complex, and 140 Na^+^ and 138 Cl^−^ ions were used to neutralize the system. The systems were equilibrated in the NVT ensemble (*T* = 300 K) for 200 ps, followed by a 500 ps simulation carried out in the NPT ensemble (*T* = 300 K, *P* = 1 atm). The GROMACS^30^ program was employed for the MD simulations. In the sMD simulations, the force field was scaled by a factor of 0.4. Heavy atoms of the protein backbone were restrained with a weak harmonic potential (with the force constant *k* = 50 kJ mol^−1^ nm^−2^) except for residues within 6 Å of ASEM and its analogues. The unrestrained residues are Ser32–Ser34, Leu36, Phe52–Gln55, Ala89, Tyr91, Thr101–Pro102, Leu106, Leu116–S118, Gln143–His148, Glu158–Asp160, Ser162–Gly163, Arg182–Asp193, and Phe196. For each system, twenty simulation runs were performed with the scaled force field. Each simulation was stopped once the ligand was fully unbound from the pocket. The residence times thus obtained as the characteristic parameter of the unbinding times of each simulation run. The mass of the ^18^F isotope of [^18^F]ASEM is not considered in the simulations.

### *In Vitro* binding assay

The 14 compounds calculated and tested in this work were commercially synthesized by Piramal Pharma solution, Ahmedabad India. Purity was checked by HPLC and ^1^H NMR. All compounds hold a purity of 95%. The compounds were thus diluted in DMSO to a concentration of 10 mM. Proportions of 50 μl aliquots of the 10 mM solution were dispensed into plastic vials which were kept frozen at −20 °C until sent to Cerep, Eurofins, France for measurement of α7 nicotinic receptor activity. The molecular weight of each compound was calculated and provided to Cerep. Before analysis each tube with compound was thawed and a solution of 100 nM was prepared for each compound. The test system consisted of an *in vitro* binding study was performed using human neuronal α7 transfected neuroblastoma cells SH-SY5Y cells (human recombinant) incubated with 0.05 nM ^125^I-α-bungarotoxin and 100 nM test compound at 37 °C for 120 minutes. The value of bound radioactivity was calculated with a scintillation counter. The inhibition was calculated as the percentage of displacement of ^125^I-α-bungarotoxin by each compound. ±Epibatidine (IC_50_ = 94 nM, *K*_i_ = 82 nM) was used as a reference in this work.

## Funding

This work was financially supported from the Swedish Foundation for Strategic Research (SSF) through the project “New imaging biomarkers in early diagnosis and treatment of Alzheimer's disease” (Contract RB13-0192), the Swedish Science Research Council “Proteinopathies in neurodegenerative disorders-new imaging biomarkers for early disease detection and new drug targets” (A.N.: VR project no. 2017-06086).

## Author contributions

Y. Z., G. K., and J. L. performed molecular docking and FEP calculations. C. H., A. N, B. L., and Y. T. provided scientific consulting for the manuscript. H. Å. directed the projects. All the authors contributed to discussion.

## Conflicts of interest

There are no conflicts of interest.

## Supplementary Material

RA-011-D0RA10435C-s001
