# Supplementary material for: In silico studies of ASEM analogues targeting α7-nAChR and experimental verification
Source: RSC Adv. 2021 Jan 21;11(7):3942–51. doi: 10.1039/d0ra10435c (PMC9134020; doi:10.1039/d0ra10435c)
Supplement: RA-011-D0RA10435C-s001 [file RA-011-D0RA10435C-s001.pdf]

Electronic Supplementary Material (ESI) for RSC Advances.
This journal is © The Royal Society of Chemistry 2021

In silico studies of ASEM analogues targeting α7-nAChR and experimental verification

Yang Zhou[a], Guanglin Kuang[b], Junhao Li[b], Christer Halldin[c], Agneta Nordbergc[d,e], Bengt Långström[f], Yaoquan Tu[b], and Hans Ågren[a,g]*

[a]*Department of Physics and Astronomy, Uppsala University, Box 516, SE-751 20 Uppsala, Sweden*

[b]*Division of Theoretical Chemistry and Biology, School of Biotechnology, Royal Institute of Technology (KTH), AlbaNova University Center, S-106 91, Stockholm, Sweden. E-mail: hagren@kth.se*

[c]*Karolinska Institutet, Department of Clinical Neuroscience, Centre for Psychiatric Research, 171 76, Stockholm, Sweden*

[d]*Department of Neurobiology, Care Sciences and Society, Division of Clinical Geriatrics, Center for Alzheimer Research, Neo 141 84 Stockholm and* [e]*Theme Aging Karolinska University Hospital, S-141 86, Stockholm, Sweden*

[f]*Department of Chemistry, Uppsala University,SE- 751 23 Uppsala, Sweden*

[g]*College of Chemistry and Chemical Engineering, Henan University, Kaifeng, Henan 475004, P.R. China*

Supporting Information

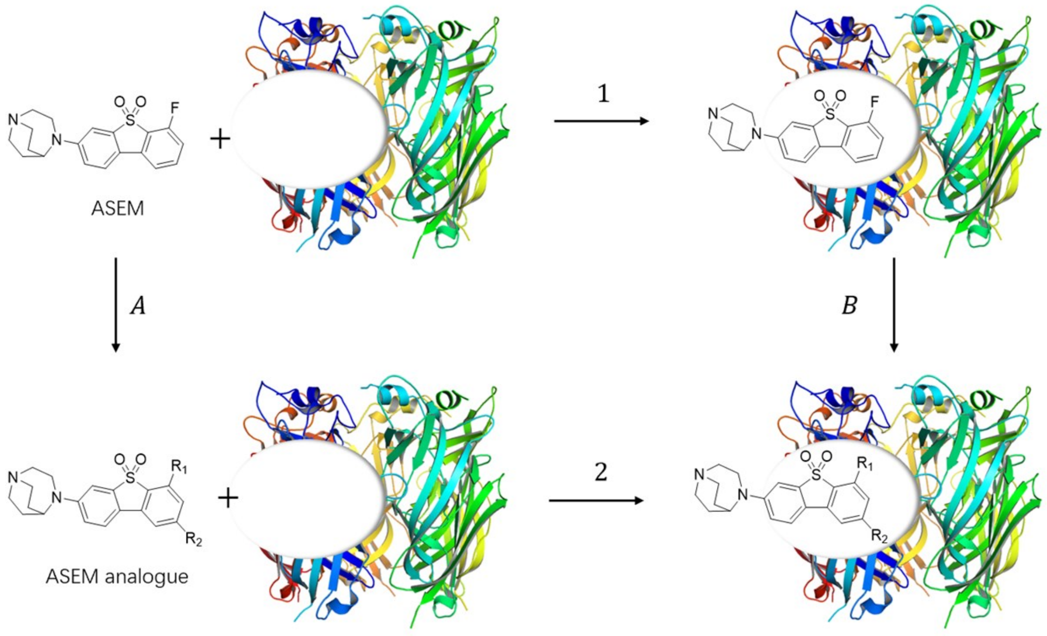

$$\Delta\Delta G = \Delta G_2 \text{-} \Delta G_1 = \Delta G_B - \Delta G_A$$

Figure S1. Illustration of the thermodynamic cycle, together with the perturbation pathways, used by an FEP+ calculation. The perturbation pathways are represented by arrows A and B. The difference in the binding free energy ($\Delta\Delta G$) between an ASEM analogue (ΔG_2) and ASEM (ΔG_1) is related to the free energy of transforming ASEM to its analogue in the solvent (ΔG_A) and in α7-AChBP (ΔG_B).
